# Isolation and molecular characterisation of *Achromobacter* phage phiAxp-3, an N4-like bacteriophage

**DOI:** 10.1038/srep24776

**Published:** 2016-04-20

**Authors:** Yanyan Ma, Erna Li, Zhizhen Qi, Huan Li, Xiao Wei, Weishi Lin, Ruixiang Zhao, Aimin Jiang, Huiying Yang, Zhe Yin, Jing Yuan, Xiangna Zhao

**Affiliations:** 1 College of Food Science, Henan Institute of Science and Technology, Xinxiang, 453003, China; 2 College of Food Science, South China Agricultural University, Guangzhou, 510642, China; 3 Qinghai Institute for Endemic Disease Prevention and Control of Qinghai Province, Xining, 810000, China; 4 Institute of Disease Control and Prevention, Academy of Military Medical Sciences, Beijing, 100071, China; 5 State Key Laboratory of Pathogen and Biosecurity, Beijing Institute of Microbiology and Epidemiology, Beijing, 100071, China

## Abstract

*Achromobacter xylosoxidans*, an opportunistic pathogen, is responsible for various nosocomial and community-acquired infections. We isolated phiAxp-3, an N4-like bacteriophage that infects *A. xylosoxidans*, from hospital waste and studied its genomic and biological properties. Transmission electron microscopy revealed that, with a 67-nm diameter icosahedral head and a 20-nm non-contractile tail, phiAxp-3 has features characteristic of *Podoviridae* bacteriophages (order *Caudovirales*). With a burst size of 9000 plaque-forming units and a latent period of 80 min, phiAxp-3 had a host range limited to only four *A. xylosoxidans* strains of the 35 strains that were tested. The 72,825 bp phiAxp-3 DNA genome, with 416-bp terminal redundant ends, contains 80 predicted open reading frames, none of which are related to virulence or drug resistance. Genome sequence comparisons place phiAxp-3 more closely with JWAlpha and JWDelta *Achromobacter* phages than with other N4 viruses. Using proteomics, we identified 25 viral proteins from purified phiAxp-3 particles. Notably, investigation of the phage phiAxp-3 receptor on the surface of the host cell revealed that lipopolysaccharide serves as the receptor for the adsorption of phage phiAxp-3. Our findings advance current knowledge about *A. xylosoxidans* phages in an age where alternative therapies to combat antibiotic-resistant bacteria are urgently needed.

*Achromobacter xylosoxidans* is a medically important opportunistic pathogen frequently associated with various nosocomial and community-acquired infections^1^. Infections caused by *A. xylosoxidans* result in significant morbidity and mortality in debilitated individuals^2^. *A. xylosoxidans*, an emerging and major pathogen that attacks people with cystic fibrosis^3^, also has the potential to cause serious infections in premature babies^4^. One of the main threats from *A. xylosoxidans* is the high rate of resistance it has to antibiotics. This species exhibits innate resistance to many antibiotic types, including cephalosporins (except ceftazidime), aztreonam, and aminoglycosides^5^. In the last few years, numerous cases of multidrug-resistant *A. xylosoxidans* infections have been documented in immunocompromised and cystic fibrosis patients^6^, and this has complicated the treatment of such infections. Little is known about the optimal therapy for *A. xylosoxidans*. In addition to the known intrinsic antibiotic resistance patterns in *A. xylosoxidans*, acquired resistance is also widely reported for this bacterium^7^. One potential option to combat *A. xylosoxidans* is use of bacteriophages^8^. Biocontrol using phages can be applied through food, agriculture, and medical fields^9^. Phages have higher bacterial specificity than antibiotics and have the advantage of minimal impact on commensal bacteria in the host^10^. Accordingly, phages that specifically target *A. xylosoxidans* may be a good choice for the control of *A. xylosoxidans* infections, especially for antibiotic-resistant *A. xylosoxidans* since avoiding an antibiotic treatment would avoid the spread of multiresistant bacteria^11^. Additionally, phages play an important role in bacterial evolution and microbial ecology^12^. The genes and activities of phages are suggested to be a driving force in maintaining genetic diversity of the bacterial community^13^. To date, however, only a few *A. xylosoxidans*-specific phages have been studied in detail^14^, there are only three reported fully sequenced *A. xylosoxidans* phages, including phiAxp-1 (GenBank accession number KP313532)^15^, JWAlpha (KF787095)^14^ and JWDelta (KF787094)^14^. Thus, isolating and characterizing new *A. xylosoxidans* phages is an essential prerequisite for developing efficient biocontrol agents against *A. xylosoxidans*. Herein, we isolated and genome sequenced a virulent *A. xylosoxidans* bacteriophage (phiAxp-3) of the *Podoviridae* family and identified its receptor. We also investigated the effect of various physicochemical treatments on phage stability.

## Results and Discussion

### Morphology and host range

**Figure 1:**
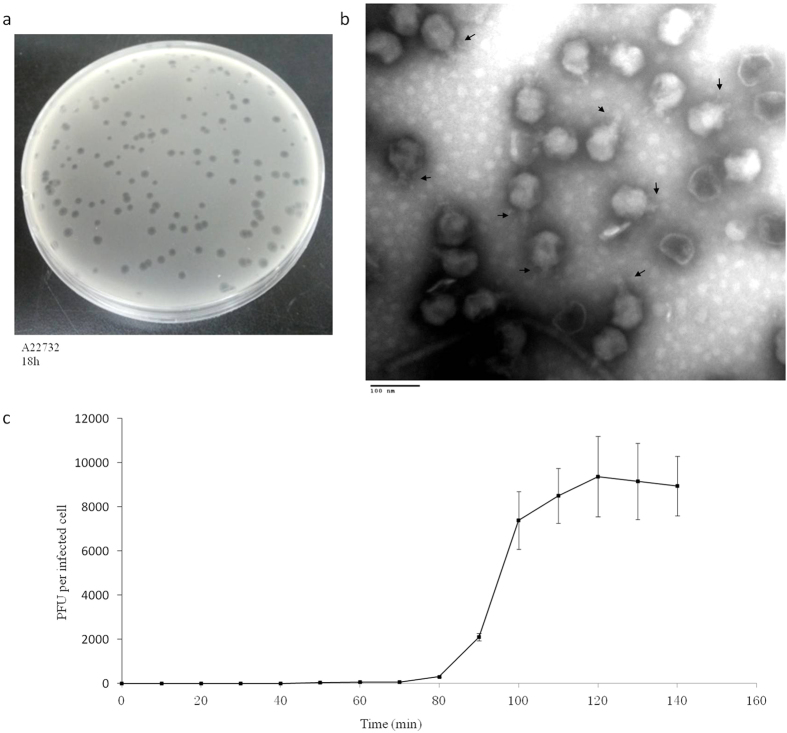
Isolated *Achromobacter* phage phiAxp-3. (**a**) Plaque morphology of phage phiAxp-3. (**b**) Transmission electron micrographs of phiAxp-3. Arrows indicate the short noncontractile tails. Phage particles were negatively stained with 2% phosphotungstic acid. Scale bar, 100 nm. (**c**) One-step growth curves for phiAxp-3 with *A. xylosoxidans* strain A22732. Plaque-forming units per ml of A22732 culture at different time points. Each time point represents the mean value of three experiments.

Phage phiAxp-3 was isolated from raw hospital sewage in China, using the *A. xylosoxidans* A22732 strain as the host; this bacterium produces OXA-114e and IMP-1 carbapenemases, which confer resistance to multiple β-lactam antibiotics including carbapenems^16^. Phage phiAxp-3 formed round plaques with transparent centres on double-layer plates (Fig. 1a). Transmission electron microscopy of the phiAxp-3 particles showed that phiAxp-3 possesses an isometric head with a diameter of about 67 nm and a short tail with an approximate length of 20 nm (Fig. 1b), thereby matching the typical morphological features of *Podoviridae* family viruses. Host range testing suggested that phiAxp-3 was able to successfully infect all *A. xylosoxidans* strains tested, unlike other species that were tested (Table 1). Besides the A22732 strain, which is reported to be multidrug-resistant^16^, all three of the other clinical *A. xylosoxidans* strains investigated here have been shown to be resistant to aztreonam and tobramycin^15^.

### One-step growth curve

We performed a one-step growth curve experiment for phiAxp-3 to determine its latent time period and phage burst size. Burst size and latent period in phages are influenced by the host, the composition of the growth medium, the incubation temperature and the specific growth rate^17^. The latent period for phiAxp-3 was about 80 min, after which there was a gradual increase in the number of viral particles released (Fig. 1c). It took about 40 min for the phages to reach the growth plateau phase and this resulted in burst sizes of ca. 9000 plaque-forming units (PFU) per infected cell.

### Phage stability

**Figure 2:**
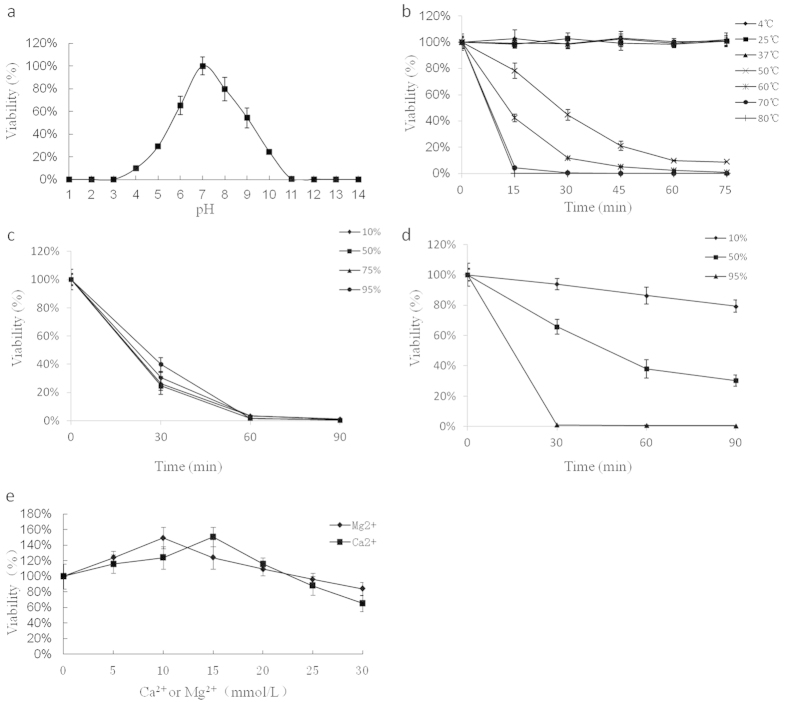
Resistance of phage phiAxp-3 to physical and chemical agents. (**a**) The effect of pH on the adsorption of phage phiAxp-3 to *A. xylosoxidans* A22732 in LB broth. (**b**) Inactivation kinetics of phage phiAxp-3 at 4 °C, 25 °C, 37 °C, 50 °C, 60 °C, 70 °C and 80 °C. (**c**) Inactivation kinetics of phage phiAxp-3 in the presence of 10%, 50%, 75% and 95% ethanol. (**d**) Inactivation kinetics of phage phiAxp-3 in the presence of 10%, 50% and 95% isopropanol. (**e**) Effect on phage phiAxp-3 titre of incubation in LB broth with and without CaCl_2_ or MgCl_2_ (0, 5, 10, 15, 20, 25 and 30 mmol/l) at 37 °C. For all the graphs, the values represent the mean of three determinations.

Figure 2a shows the pH sensitivity of phage phiAxp-3. The phage titres decreased to different extents when the pH was above or below 7. At pH 4 and pH 10, reductions of 90.25% and 75.76% in phage particle counts were observed, respectively. Almost no viral particles were detected at pH 1 and pH 14. The viability loss when phiAxp-3 was subjected to temperatures of 25 °C, 37 °C, 50 °C, 60 °C, 70 °C and 80 °C is shown in Fig. 2b. A control at temperature of 4 °C was also included. The phage titres reduced dramatically at 50 °C, 60 °C, 70 °C and 80 °C. After 75 min at 50 °C, the phage titre reduced by 91.2%. At 80 °C, a 99.86% reduction in viral particles was recorded after 15 min, and compared with the control, after 75 min only 0.0002% of the viral particles were detected. Scarcely any reduction in the phage titres were observed at 4 °C, 25 °C and 37 °C after 75 min of treatment. The survivor curves for phiAxp-3 in different biocides are shown in Fig. 2c,d. The results show that the presence of ethanol at low (10%) and high (95%) concentrations reduced the phage titres (Fig. 2c). The phage titres reduced by 20.75%, 69.76% and 99.62% after 75 min of treatment with isopropanol at 10%, 50% and 95%, respectively (Fig. 2d). Divalent ions such as Ca^2+^ or Mg^2+^ are necessary for phage attachment and intracellular growth^18^. phiAxp-3 showed divalent cation dependency for plaque development, but the concentration of Ca^2+^ or Mg^2+^ had to be less than or equal to 20 mM (Fig. 2e).

### Genomic features of bacteriophage phiAxp-3

**Figure 3:**
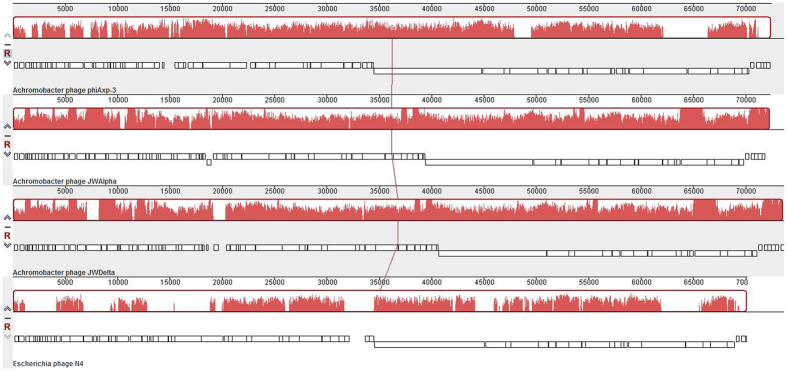
Multiple genome alignment generated by Mauve software (http://asap.ahabs.wisc.edu/mauve/), and the chromosomes of *Achromobacter* phages phiAxp-3, JWAlpha and JWDelta and the *Enterobacter* phage, N4. Genome similarity is represented by the height of the bars, which correspond to the average level of conservation in that region of the genome sequence. Completely white regions represent fragments that were not aligned or contained sequence elements specific to a particular genome.

Analysis of a bacteriophage’s genome is an important preliminary step towards the development of phage therapy^19^. Whole-genome sequencing and assembling of the phiAxp-3 genome generated a circular molecule of 72,409 bp in size. The assembly was terminally permuted but not redundant after the original sequencing was completed. An initial whole genome Basic Local Alignment Search Tool (BLAST) analysis of phiAxp-3 against the National Center for Biotechnology Information database and multiple genome alignments showed that phiAxp-3 is related to two N4-like viruses (i.e., JWAlpha and JWDelta), indicating that phiAxp-3 is an N4-like phage (Fig. 3). It is well-known that N4-like phages have linear genomes and terminal repeats, but the terminal repeats are usually not identical^20^. Additionally, it is important to verify experimentally the ends of the phage genome rather than relying on genome assembly programs^21^. Therefore, to determine whether the phiAxp-3 genome is linear or circularly permuted and whether the ends are fixed or variable, restriction enzyme analyses were performed. *Blp*I restriction enzyme digestion of phiAxp-3 DNA produced two distinct fragments, thereby indicating the presence of a single recognition site in the viral DNA, and four distinct fragments when cut with *Eag*I, thereby indicating the presence of three sites in the viral DNA (Supplementary Figure 1a). These findings clearly indicate that phiAxp-3 has a fixed linear genome structure without circular permutation. Terminal restriction enzyme fragments and primer walking experiments were used to determine the sequence of the phiAxp-3 genomic ends. The 5′ genomic end was predicted to be contained within a 3.5 kb *Blp*I fragment (Supplementary Figure 1b); hence, the gel-purified *Blp*I fragment was used as a template for sequencing reactions. Primer (P1), which was designed to read off the 5′ end of the genome, exhibited a detectable drop in signal intensity, indicating that the likely 5′ genome end had been reached. Sequencing from the predicted 3′ end, using the 3′ 5.4 kb *Eag*I fragment and primer P2 (Supplementary Figure 1b), produced the repeat region at the 3′ end. Therefore, the phiAxp-3 genome has direct terminal repeats of 416 bp and possesses no cohesive ends; this result is consistent with those described previously for JWAlpha and JWDelta phages^14^. An alignment of the direct terminal repeats of phiAxp-3 is very similar to those for JWAlpha and JWDelta (Supplementary Figure 2). The terminal repeats in phiAxp-3 are 51 bp longer than that of JWAlpha (365 bp) and 4 bp shorter than that of JWDelta (420 bp). When the non-consecutive indels were removed, we found that they shared about 77% identity with those of JWAlpha and JWDelta. The additional 416-bp repeat means that the genome is 72,825-bp-long (GC content, 55.2%), rather than 72,409-bp-long (Supplementary Figure 1c).

**Figure 4:**
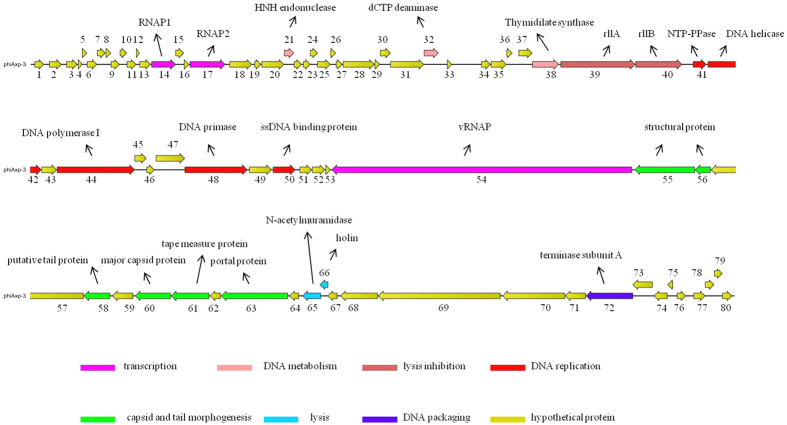
Genome map of phage phiAxp-3. Representation of the open reading frame (ORF) (ORF 1 to 80) organisation of phage phiAxp-3. The predicted genes are indicated as arrows. Blue arrows, DNA regulation module; purple arrows, packaging module; yellow arrows, phage structural proteins; red arrows, host lysis proteins; green arrows, lysis/lysogeny module; black arrows, hypothetical proteins.

The order and arrangement of the open reading frames of the revised genome are the same as the previously sequenced version and were not affected by the reorganisation of the terminal regions of the genome. A total of 80 protein coding genes were predicted in the genome and ranged from 120 to 10,287 bp, 22 of which are leftward oriented while the others are rightward oriented (Fig. 4). N4-like phages are a class of virulent *Podoviridae* phages and members of this group are lytic against their hosts^22^. The phiAxp-3 genome sequence shares 51.6% and 50.4% nucleotide identity with JWAlpha and JWDelta, respectively. The three phages were isolated from samples obtained from two locations that are geographically far apart (phiAxp-3 was isolated in China, JWAlpha and JWDelta were isolated in Germany)^14^. For comparison, phiAxp-3 shares 40.8% nucleotide identity with phage N4. JWAlpha and JWDelta share 96.6% nucleotide sequence identity. The overall architecture of N4 is shared among all phages of this group. Based on our analysis, the annotated proteins of phiAxp-3 can be categorised into the following functional groups: Transcription (RNA polymerase; RNAP1, RNAP2, vRNAP), DNA metabolism (HNH endonuclease, dCTP deaminase, thymidylate synthase), lysis inhibition (rllA, rllB), DNA replication (NTP-PPase, DNA helicase, DNA polymerase, DNA primase, ssDNA-binding protein), virion morphogenesis (structural proteins, tail protein, major capsid protein, tape measure protein, portal protein), host lysis (N-acetylmuramidase, holin) and DNA packaging (large terminase subunit) (Fig. 4). No tRNA was identified in the phiAxp-3 genome; this indicates that upon entry into the host, the phage is completely reliant on the host tRNA for its protein synthesis. Table 2 shows a detailed comparison of phiAxp-3, N4, and JWAlpha and JWDelta proteins. In our analyses, 25 phage proteins were detected using LC/ESI/MS/MS, of which 10 had annotated functions (Table 3).

### Transcription module

**Figure 5:**
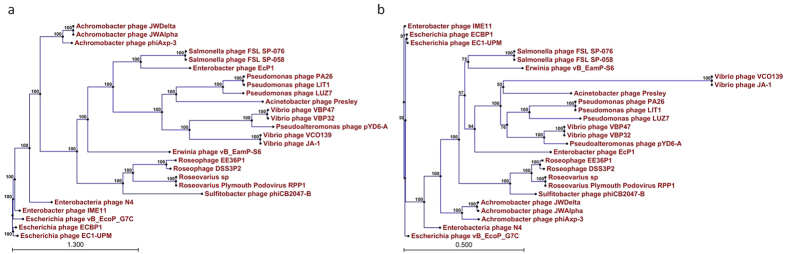
Phylogenetic tree based on the virion RNA polymerase (**a**) and large terminase subunits (**b**) of N4-like bacteriophages for which genome sequences are available. The 26 virion RNA polymerase and large terminase subunits were compared using the ClustalW program, and the phylogenetic tree was generated with the neighbour-joining method and 1000 bootstrap replicates (CLC Genomics Workbench 6).

Phage N4 employs at least three genes encoding RNAPs for the transcription of genes in different stages of its life cycle^23^. The most remarkable and highly conserved signature is the virion RNA polymerase (vRNAP), which is by far the largest protein described among all known phages^23^. vRNAP is packed into the capsid and is injected into the host cell together with phage DNA, which makes N4 the only known phage that does not depend on host RNAP for transcription of its early genes^24^. As an N4 like virus, phiAxp-3 also harbours three different RNAPs, suggesting the same transcription strategy as that used by N4. The large 3428 amino acid vRNAP (ORF54), which represents approximately 14% of the whole genome length of phiAxp-3, contains no cysteine residues. The vRNAP of phiAxp-3 shares amino acid 82% sequence identity with JWAlpha and JWDelta. Phylogenetic analysis of vRNAP from different N4 viruses revealed that phiAxp-3, JWAlpha, and JWDelta formed a separate clade from the other N4-like viruses (Fig. 5a). Besides vRNAP, phiAxp-3 possesses two different RNA polymerase subunits for transcription of phage middle genes: RNAP1 (ORF14) and RNAP2 (ORF17). RNAP1 and RNAP2 are transcribed in the opposite direction to vRNAP. In the N4 genome the RNAP1 gene is followed directly by RNAP2, but in the phiAxp-3 genome insertions of two small genes (ORF15 and ORF16) occur between RNAP1 and RNAP2. This situation differs from JWAlpha and JWDelta as they encode two RNAP2 in their genomes^14^.

### DNA metabolism

phiAxp-3 has three genes encoding proteins involved in nucleotide metabolism: an HNH endonuclease (ORF21), a deoxycytidine triphosphate (dCTP) deaminase (ORF32) and a thymidylate synthase (TS) protein (ORF38). These proteins each play a role in regulating some of the enzymes involved in DNA metabolism or replication^14^ and are similar to homologous proteins from JWAlpha, JWDelta and N4. Homing endonucleases (HEs) are able to transfer genetic elements from an HE-encoding genome to an HE-lacking recipient to promote gene recombination in phages^25^. One HE family is the HNH endonucleases, which are small DNA binding and digestion proteins characterised by two histidine residues and an asparagine residue^26^. The phiAxp-3 HNH endonuclease contains an HNH_3 domain (pfam13392) predicted to possess HNH endonuclease activity. phiAxp-3 also possesses a dCTP deaminase with a conserved dcd (PRK00416) domain. Thymidylate synthase (TS) is essential for production of dTMP and is a key enzyme involved in DNA synthesis and transcriptional regulation in organisms^27^. phiAxp-3 TS contains a Thy1 (pfam02511) domain and appears to be flavin-dependent. A thioredoxin gene that has been reported in the genomes of JWAlpha and JWDelta is absent from the phiAxp-3 genome.

### DNA replication

DNA replication genes are concentrated in a region that stretches from ORF41 to ORF50 in phiAxp-3. They are separated from the structural module by a particularly large vRNAP gene. The DNA helicase (ORF42) present in phiAxp-3 possesses greater similarity to helicases from JWAlpha and JWDelta than the helicase from N4. The same situation also exists for DNA polymerase I (ORF44), DNA primase (ORF48) and the ssDNA binding protein, ssb (ORF50). The phiAxp-3 DNA helicase contains an AAA_30 (pfam13604) domain at the N terminal and a UvrD_C_2 (pfam13538) domain at the C terminal. This DNA helicase can probably be classified within the RecD-like helicase superfamily. The DNA polymerase I present in phiAxp-3 shares 81% similarity with those present in JWAlpha and JWDelta, while Ssb is involved in DNA replication/recombination and host RNA polymerase activation in late N4 transcription^23^. It has been reported that Ssb from most N4-like viruses is located next to the DNA primase^18^. However, in the phiAxp-3, JWAlpha, JWDelta and N4 genomes, there is a gene next to the DNA primase that encodes a protein with a similar size to that of the Ssb protein (200–250 amino acids), but this protein shares no amino acid similarity with the Ssb protein.

### Virion morphogenesis

Sequence-based predictions identified the following six ORFs involved in virion morphogenesis: two phage structural proteins (ORF55 and 56), phage tail protein (ORF58), major capsid protein (ORF60), tail tape measure protein (ORF61) and portal protein (ORF63). The morphogenesis-related proteins are similar to those found in JWAlpha, JWDelta and N4. Portal proteins, which have molecular masses between 40 and 90 kDa, are not well conserved^28^. Accordingly, the phiAxp-3 portal protein is 760 amino acid residues in length, which corresponds to an 85 kDa molecular mass. Although tape measure proteins act as scaffolds for assembly of the phage-tail in *Myoviridae* and *Siphoviridae* members^23^, the presence of tape measure proteins in *Podoviridae* phages is not unusual.

### Lysis and lysis inhibition

In the phiAxp-3 genome downstream of the structurally clustered genes involved in cell lysis, we identified two ORFs located contiguously that encode a predicted *N-*acetylmuramoyl-L-alanine amidase (ORF65) and a putative phage holin (ORF66). These two proteins are required for host cell lysis and the release of new virions at the end of the lytic cycle^12^. The presence of a lysis gene but no lysogeny-related gene indicates that bacteriophage phiAxp-3 is a lytic bacteriophage. The putative amidase, predicted to be a 210-amino-acid protein, is presumably involved in cleaving the amide bond between *N*-acetylmuramoyl and the L-amino acid in peptidoglycan^12^. The predicted holin protein gene encodes a 95-amino acid molecule responsible for controlling the timing of lysis. It was assigned as a class II holin with two transmembrane domains. phiAxp-3 rIIA-like (ORF39) and rIIB-like (ORF40) proteins, which might play roles in lysis inhibition, are located upstream of the replication cluster. These types of protein were first described in phage T4, where the rI gene was found to somehow be able to detect superinfection at any point until just before the normal time of lysis and was also able to delay lysis for several hours^29^.

### DNA packaging

We were only able to identify the large subunit of the terminase (ORF72) used for DNA packaging in phiAxp-3. The large terminase subunit shares high amino acid sequence similarity with JWAlpha and JWDelta, and probably uses the same mechanism for packaging as other N4-like phages. Large terminase protein sequences have been used to construct phylogenies and decipher evolutionary relationships among phages belonging to different families^30^. Clustering of the amino acid sequences of the large terminase proteins encoded by phiAxp-3 with the other N4-like bacteriophages for which genome sequences are available^31^, clearly placed phiAxp-3 within the branch of JWAlpha and JWDelta (Fig. 5b).

### Host receptor identification

**Figure 6:**
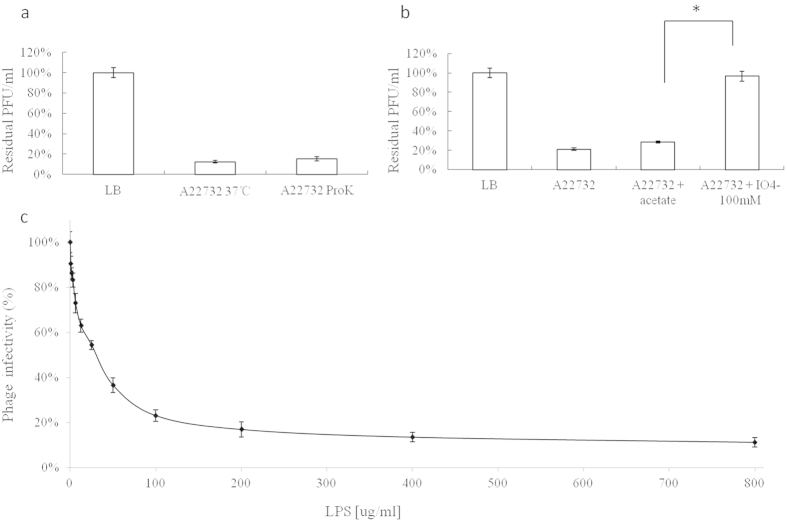
The effects of various bacterial treatments on phiAxp-3 adsorption to host cells, as determined by residual plaque-forming unit percentages. (**a**) Effect of proteinase K treatment on the adsorption of phiAxp-3 to *A. xylosoxidans* strain A22732. (**b**) Effect of periodate treatment on the adsorption of phiAxp-3 to *A. xylosoxidans* strain A22732. The control (LB and “A22732 + acetate”), untreated strain (A22732), and treatment (“A22732 + ProtK” for proteinase K treatment and “A22732+IO^4−^” for periodate treatment) groups were tested for adsorption as indicated by the *x* axes. Error bars denote statistical variations. Statistical significance was determined by a Student *t* test for comparison between the treated and untreated groups. *P 0.05. (**c**) Inactivation of phage phiAxp-3 by lipopolysaccharide derived from *A. xylosoxidans* A22732. The percentage infectivity was determined after 1 h of incubation at 37 °C. Error bars denote statistical variations.

Phage infection is dependent on the presence of an attachment site on the host cell surface and any exposed component of the cell surface can potentially act as a receptor^32^. As a Gram-negative bacterium, the exposed surface of *A. xylosoxidans* consists essentially of a complex of lipopolysaccharide (LPS) and proteins^32^. Thus it is important to determine whether LPS and proteins are recognisable by phages during infection. To identify the host receptor for phiAxp-3, the outer membrane proteins and the carbohydrate structure of the *A. xylosoxidans* cell surface were destroyed by proteinase K and periodate, respectively (Fig. 6a,b). The results revealed that the absence of carbohydrate structure inhibits phage propagation, suggesting that phiAxp-3 uses the bacterial LPS layer as its specific receptor. The results were confirmed by the phage inactivation assay performed with pure LPS isolated from strain A22732. The experiments revealed a direct correlation between LPS concentration and inhibition of viral particle infectivity (Fig. 6c). LPS at 25 μg per ml was needed to inhibit the activity of 3.2 × 10^4^ pfu phiAxp-3 by 50%, while LPS at 800 μg per ml resulted in 89% inactivation of phiAxp-3.

### Concluding remarks

In this study, we have presented the characteristics of phiAxp-3, a lytic phage that was found to infect clinical isolates of *A. xylosoxidans*. We propose that phiAxp-3 is assigned to the *Caudovirales* order (*Podoviridae* phage family) based on its morphological features and genomic characteristics. Characterisation and analysis of genome structure and gene function are necessary steps before bacteriophages can be approved as therapeutic agents. According to the overall genomic organisation and sequence similarities revealed herein, we suggest that phiAxp-3 is classified as the N4-like phage group. In its 72,825 bp linear DNA genome, phiAxp-3 has fixed ends with direct terminal repeats of 416 bp. Phage phiAxp-3 is genetically related to the N4-like phages JWAlpha and JWDelta, and phylogenetic analysis of its RNAPs and large terminase subunits supports this assignment.

The phage infection process begins with the adsorption of the phage to the bacterial receptor, which is present on the cell surface^33^. Exploration of the receptors used by phages is essential for understanding the processes underlying phage lysis and for research on phage therapy. Analysis of the phiAxp-3 putative cell wall receptor revealed that phiAxp-3 recognises LPS as its primary receptor for adsorption, thereby accounting for the specificity of its interactions with its host bacterium. Although bacteria can develop resistance to their viral predators, finding new phages that can kill drug-resistant bacteria is not difficult, because phages continually evolve alongside mutated bacteria^10^. LPS acts as an important virulence factor for *A. xylosoxidans*^34^, and receptor mutated strains will be avirulent or attenuated. Furthermore, phage cocktails, containing different types of phages, can effectively prevent bacteria from developing resistance to phages^10^. Facing the emerging threat from multi-drug resistance *A. xylosoxidans*, the lytic power of phiAxp-3 combined with its specificity for *A. xylosoxidans* makes phiAxp-3 an appealing agent for therapeutic or disinfection applications.

## Methods

### Bacterial strains and growth conditions

All bacterial strains (including the phage indicator strain and the strains used for host range identification) were grown at 37 °C in Luria-Bertani (LB) broth. To isolate and purify the phages, the *A. xylosoxidans* strain A22732 was used as an indicator strain to reveal the presence of phages in the hospital sewage collected from the Second Artillery General Hospital of Chinese People’s Liberation Army (Beijing, China), using the double agar overlay plaque assay described previously for the isolation of lytic phages^35^. Plaques picked from agar plates were placed in 5 ml of LB broth and incubated with 0.3 ml of an overnight culture of the host strain. Incubation at 37 °C was performed until lysis of the culture was complete. The host range of the phages were examined using 35 clinical strains of different bacterial species stored at our microorganism centre using standard spot tests^36^.

### Transmission electron microscopy

One drop of purified phiAxp-3 particles was adsorbed to a 230-mesh Formvar/carbon-coated copper grid for several minutes, followed by staining with 2% (wt/vol) phosphotungstic acid (pH 7). Samples were examined with a Philips EM 300 electron microscope operated at 80 kV.

### One-step growth curve

Mid-exponential growing cultures of *A. xylosoxidans* A22732 cells were harvested and suspended in LB broth. Phages were added at a multiplicity of infection of 0.1. At 10-min intervals over 140 min, aliquots from each dilution were collected for phage counts^37^. Latent period, burst time and burst size were calculated from the one-step growth curve, as described previously^38^. Measurement of the duration of a phage’s latent-period was accomplished by detecting the delay between phage adsorption of a bacterium and the liberation of phage virions^39^,^40^,^41^. We calculated the burst size from the ratio of the final count of liberated phage particles to the initial count of infected bacterial cells during the latent period.

### Stability studies

We assayed phage stability in LB broth at pH values ranging from 1 to 14, after incubation for 60 min at 37 °C, and the phages that survived were diluted and counted immediately. Seven temperatures (4, 25, 37, 50, 60, 70 and 80 °C) were selected to study the thermal tolerance of phiAxp-3 in LB broth at 15-min sampling intervals. Biocide resistance was determined using the common biocides ethanol (10%, 50%, 75% and 95% v/v) and isopropanol (10%, 50% and 95%) at 30-min intervals for sampling. The influence of Ca^2+^ and Mg^2+^ on phage lysis was investigated by incubation (37 °C) of infected *A. xylosoxidans* A22732 in LB agar with and without CaCl_2_ or MgCl_2_ (0, 5, 10, 15, 20, 25 and 30 mmol/l). Plaque formation was investigated using the double-layer plate technique. We expressed the results as a percentage of the initial viral counts.

### DNA isolation and genome sequencing

Genomic DNA was extracted from purified phage particles with phenol-chloroform (24:1, vol/vol) method described previously^15^. Whole-genome sequencing of the phiAxp-3 phage was performed with an Illumina HiSeq2500 sequencer. The reads were assembled using the CLC genomics Workbench *de novo* assembly algorithm (CLC bio, Cambridge, MA). The BLASTP program was used to search putative homologies and proteins sharing similarities with predicted phage proteins (http://www.ncbi.nlm.nih.gov/BLAST/). Sequence alignment and phylogenetic analysis were performed using ClustalW (Slow/Accurate, IUB) and Mauve software (http://asap.ahabs.wisc.edu/mauve/). LC/ESI/MS/MS spectra (Q-TOF Ultima API, Micromass UK Ltd.) were used to identify phage proteins, as described previously^42^.

### Structure determination of phiAxp-3 genome ends

The physical genome structure (linear vs circular) of phiAxp-3 was assessed by analysing its restriction digestion profiles using the conditions recommended by the manufacturer (New England Biolabs, Ipswich, MA). Lambda DNA/*Hin*dIII Markers (Thermo Scientific, Waltham, MA) were used for estimating DNA fragment sizes. Phage phiAxp-3 genome-end fragments resulting from digestion with *Blp*I or *Eag*I restriction enzymes were excised from agarose gels and extracted using a QIAquick Gel Extraction Kit (Qiagen, Hilden, Germany). The DNA fragments isolated were checked for purity by electrophoresis and used as templates for sequencing.

### Receptor identification

The receptor properties of phiAxp-3 were determined as described previously^43^. Briefly, *A. xylosoxidans* A22732 cultures were treated with proteinase K (0.2 mg/ml; Promega) at 37 °C for 3 h and sodium acetate (50 mM, pH 5.2) containing 100 mM IO^4−^ at room temperature for 2 h (protected from light) to determine whether proteinase K or periodate could destroy the phage receptor. Next, a phage adsorption assay was performed as described previously^44^. LPS extraction from *A. xylosoxidans* cultures was performed using an LPS extraction kit from Intron Biotechnology (17144; Boca Scientific, Boca Raton, FL) according to the manufacturer’s instructions. Phage inactivation by LPS was performed as described previously^45^.

### Nucleotide sequence accession number

The annotated genome sequence for the phage phiAxp-3 was deposited in the NCBI nucleotide database under the accession number KT321317.

## Additional Information

**How to cite this article**: Ma, Y. *et al*. Isolation and molecular characterisation of *Achromobacter* phage phiAxp-3, an N4-like bacteriophage. *Sci. Rep.* **6**, 24776; doi: 10.1038/srep24776 (2016).

## Supplementary Material

Supplementary Information

## Figures and Tables

**Table 1:** Host range infection of the phage phiAxp-3. ^−^absent; ^+^present.

Species	ID	Infection
*Achromobacter xylosoxidans*	A22732	+
*A. xylosoxidans*	5271	+
*A. xylosoxidans*	844	+
*A. xylosoxidans*	6065	+
*Enterobacter aerogenes*	3-SP	−
*E. aerogenes*	201316724	−
*E. aerogenes*	2015-301	−
*E. aerogenes*	13208	−
*E. aerogenes*	A29864	−
*E. aerogenes*	A36179	−
*Escherichia coli*	ATCC 25922	−
*E. coli*	DH10B	−
*E. coli*	EC600	−
*Klebsiella pneumoniae*	ATCC BAA-1706	−
*K. pneumoniae*	ATCC BAA-2146	−
*K. pneumoniae*	ATCC BAA-1705	−
*K. pneumoniae*	K2044	−
*K. pneumoniae*	511	−
*Serratia marcescens*	wk2050	−
*S. marcescens*	201315732	−
*S. marcescens*	wj-1	−
*S. marcescens*	wj-2	−
*S. marcescens*	wj-3	−
*E. cloacae*	T5282	−
*E. cloacae*	TI3	−
*E. sakazakii*	45401	−
*E. sakazakii*	45402	−
*Leclercia adcarboxglata*	P10164	−
*Raoultella ornithinolytica*	YNKP001	−
*Stenotrophomonas maltophilia*	9665	−
*Citrobacter freundii*	P10159	−
*Vibrio parahaemolyticus*	J5421	−
*Pseudomonas aeruginosa*	PA01	−
*Acinetobacter baumannii*	N1	−
*Shigella sonnei*	#1083	−

**Table 2:** phiAxp-3 gene annotations.

ORFs	Start	End	Strand	Length (aa)^a^	Vs			Function	Conserved Protein Domain Family
					JWAlpha	JWDelta	N4		
ORF01	141	461	+	106	73	73	51		
ORF02	640	1065	+	141	67	67	43		
ORF03	1229	1606	+	125					
ORF04	1628	1774	+	48					
ORF05	1767	1937	+	56					
ORF06	1934	2284	+	116	41	41			
ORF07	2281	2571	+	96	28	28			
ORF08	2574	2759	+	61					
ORF09	2752	3057	+	101	72				
ORF10	3061	3300	+	79	50	50			
ORF11	3297	3623	+	108	55				
ORF12	3620	3739	+	39					
ORF13	3736	4119	+	127	71	73	55		
ORF14	4142	4969	+	275	80	80	62	RNA polymerase 1	PHA00452; COG5108
ORF15	4966	5253	+	95					
ORF16	5256	5435	+	59					
ORF17	5462	6676	+	404	83		55	RNA polymerase 2	pfam00940; PHA00452
ORF18	6814	7587	+	257	49	41			
ORF19	7674	7901	+	75	54	45			
ORF20	7916	8689	+	257	42	44			
ORF21	8689	9027	+	112	79	80	64	HNH endonuclease	pfam13392
ORF22	9024	9290	+	88					
ORF23	9340	9594	+	84	44	44			
ORF24	9578	9841	+	87	74	76			
ORF25	9823	10278	+	151	66	77			
ORF26	10275	10463	+	62	64				
ORF27	10465	10686	+	73	39	40			
ORF28	10704	11786	+	360	73	73	51		
ORF29	11789	11974	+	61					
ORF30	11978	12340	+	120	46	46			
ORF31	12325	13482	+	385	80	80	57		
ORF32	13479	13976	+	165	74	75	58	dCTP deaminase	cd07557; COG0717; PRK00416; TIGR02274; PHA01707
ORF33	14274	14435	+	53	53	53			
ORF34	15442	15765	+	107	70	69			
ORF35	15776	16309	+	177	24	27			
ORF36	16309	16509	+	66	47				
ORF37	16724	17191	+	155	67				
ORF38	17188	18099	+	303	78		54	Thymidilate synthase	pfam02511; TIGR02170; COG1351; PRK00847
ORF39	18162	20717	+	851	61	61	28	rIIAlike protein	cd00075; pfam13589; smart00387; COG1389; PRK04184; TIGR01052
ORF40	20727	22319	+	530	69	68	33	rIIBlike protein	
ORF41	22695	23153	+	152	87	86	45	NTP pyrophosphohydrolase	cd11530; COG4696; pfam01503
ORF42	23208	24515	+	435	79	80	56	DNA helicase	pfam13604; pfam13538; pfam13086; COG0507; TIGR01448; PRK13826; PRK10875
ORF43	24525	25046	+	173	65	65	40		
ORF44	25055	27715	+	886	81	81	63	DNA polymerase I	pfam00476; cd08637; smart00482; smart00474; COG0749; TIGR00593; PRK05755
ORF45	27712	28116	+	134	66	66	49		
ORF46	28119	28394	+	91	71	75	59		
ORF47	28440	29432	+	330	82	82	58		
ORF48	29429	31576	+	715	91	91	76	DNA primase	pfam08708; smart00942
ORF49	31640	32407	+	255	85	85	67		
ORF50	32450	33217	+	255	64	64	49	ssDNA binding protein	
ORF51	33368	33781	+	137	83		52		
ORF52	33792	34235	+	147	63		58		
ORF53	34252	34431	+	59	58				
ORF54	34464	44750		3428	82	82	55	RNA polymerase	PRK10811; COG0810
ORF55	44849	46900		683	70	70	34	structural protein	
ORF56	46913	47446		177	69		68	structural protein	
ORF57	47459	50110		883	54	54	46		
ORF58	50114	51013		299	77		65	putative tail protein	
ORF59	51090	51806		238	68	68	55		
ORF60	51873	53096		407	94	95	84	major capsid protein	TIGR04387
ORF61	53109	54413		434	79		53	tape measure protein	
ORF62	54439	54813		124	54		39		
ORF63	54824	57106		760	86	87	66	portal protein	
ORF64	57162	57485		107					
ORF65	57608	58240		210	90	93	62	Nacetylmuramidase	pfam05838; pfam09374; COG3926
ORF66	58203	58490		95	85	76	31	putative holin protein	
ORF67	58469	58804		111	68	63	55		
ORF68	58909	60186		425			69		
ORF69	60206	64393		1395			54	160 kDa protein	
ORF70	64462	66606		714	40	40	39		
ORF71	66610	67314		234	61	61	54		
ORF72	67322	68932		536	87	87	76	terminase subunit A	TIGR01630; pfam03237
ORF73	68925	69605		226	90	88	65		
ORF74	69649	70116		155	65	66			
ORF75	70113	70304		63					
ORF76	70440	70751	+	103	29	29	30		
ORF77	71010	71411	+	133					
ORF78	71401	71718	+	105					
ORF79	71715	71999	+	94	46				
ORF80	71996	72328	+	110	42				

^a^amino acids.

**Table 3:** Virion proteins detected by LC/ESI/MS/MS.

Protein ID	Theoretical avg. mass (Da)	Score	Matches	Sequences	Annotated Function
GI:921956017	15700	5256	107 (103)	13 (12)	Hypothetical protein
GI:921956033	26713	19132	296 (290)	19 (18)	Hypothetical protein
GI:921956035	29169	1309	35 (31)	14 (14)	Hypothetical protein
GI:921956040	17465	169	3 (3)	3 (3)	Hypothetical protein
GI:921956043	40735	25	1 (1)	1 (1)	Hypothetical protein
GI:921956046	44368	30	2 (2)	2 (2)	Hypothetical protein
GI:921956053	34122	152	3 (3)	3 (3)	Thymidilate synthase
GI:921956054	96533	198	7 (5)	6 (5)	Hypothetical protein
GI:921956055	57942	105	4 (3)	4 (3)	rIIB like protein
GI:921956062	38520	41	2 (1)	2 (1)	Hypothetical protein
GI:921956064	29341	185	6 (4)	5 (3)	Hypothetical protein
GI:921956065	27758	136	4 (4)	4 (4)	ssDNA-binding protein
GI:921956069	369998	3409	70 (61)	47 (41)	virion RNA polymerase
GI:921956070	72167	16474	268 (258)	44 (43)	Structural protein
GI:921956071	17648	1468	38 (38)	6 (6)	Structural protein
GI:921956072	98259	68	2 (2)	2 (2)	Hypothetical protein
GI:921956045	32808	2670	53 (53)	15 (15)	Putative tail protein
GI:921956074	26168	750	15 (15)	9 (9)	Hypothetical protein
GI:921956075	44531	18929	285 (274)	32 (32)	Major capsid protein
GI:921956077	14174	149	4 (3)	4 (3)	Hypothetical protein
GI:921956078	84941	732	20 (19)	5 (5)	Portal protein
GI:921956083	49043	592	13 (11)	10 (8)	Hypothetical protein
GI:921956084	157480	293	5 (5)	5 (5)	160 kDa protein
GI:921956085	76176	1632	33 (30)	25 (23)	Hypothetical protein
GI:921956086	26536	4608	103 (92)	15 (14)	Hypothetical protein
