# Supplementary material for: Isolation and molecular characterisation of Achromobacter phage phiAxp-3, an N4-like bacteriophage
Source: Sci Rep. 2016 Apr 20;6:24776. doi: 10.1038/srep24776 (PMC4837373; doi:10.1038/srep24776)
Supplement: Supplementary Information [file srep24776-s1.pdf]

Isolation and molecular characterisation of *Achromobacter* phage phiAxp-3, an N4-like bacteriophage

Yanyan Ma[1#], Erna Li[2#], Zhizhen Qi[3#], Huan Li[4], Xiao Wei[4], Weishi Lin[4], Ruixiang Zhao[1], Aimin Jiang[2], Huiying Yang[5], Zhe Yin[5]*, Jing Yuan[4]*, Xiangna Zhao[4]*

[1]College of Food Science, Henan Institute of Science and Technology, Xinxiang, China, 453003

[2]College of Food Science, South China Agricultural University, Guangzhou, China , 510642

[3]Qinghai Institute for Endemic Disease Prevention and Control of Qinghai Province, Xining, China, 810000

[4]Institute of Disease Control and Prevention, Academy of Military Medical Sciences, Beijing, China, 100071

[5]State Key Laboratory of Pathogen and Biosecurity, Beijing Institute of Microbiology and Epidemiology, Beijing, China, 100071

[#]: These authors contributed equally to this work.

*Correspondence: Zhe Yin, State Key Laboratory of Pathogen and Biosecurity, Beijing Institute of Microbiology and Epidemiology, Beijing, China; Jing Yuan, Institute of Disease Control and Prevention, Academy of Military Medical Sciences, Beijing, China; Xiangna Zhao, Institute of Disease Control and Prevention, Academy of Military Medical Sciences, Beijing, China

Tel: +86-10-66948434; Fax: +86-10-66948301

E-mail: jerry9yin@163.com; yuanjing6216@163.com; xnazhao@163.com

Supplementary Figure legends

Supplementary Figure 1. Schematic illustration of the structure and linearity of phiAxp-3 genomic ends. (a) Restriction fragment length polymorphism analysis of phiAxp-3 DNA. Genomic DNA from phage phiAxp-3 was digested with the enzymes indicated (*Blp*I and *Eag*I) and run on an agarose gel (0.7%). (b) Sequence analysis of the ends of the phiAxp-3 genome. Black arrows indicate the oligonucleotide primers (P1 and P2) that were used, and the line with two arrowheads indicates the end fragments produced by genome digestion with *Blp*I and *Eag*I restriction enzymes. Genome numbering of the sequences is that of the revised sequence. The repeat regions are shown in black at both ends of the genome. (c) Diagram showing the arrangement of the phiAxp-3 genome as originally sequenced and the revised genome arrangement.

Supplementary Figure 2. Multiple sequence alignment of the direct terminal repeats of phage phiAxp-3 with JWAlpha and JWDelta. The alignment was performed using ClustalX. Positions with a single, fully conserved base pair are indicated by an asterisk (*).

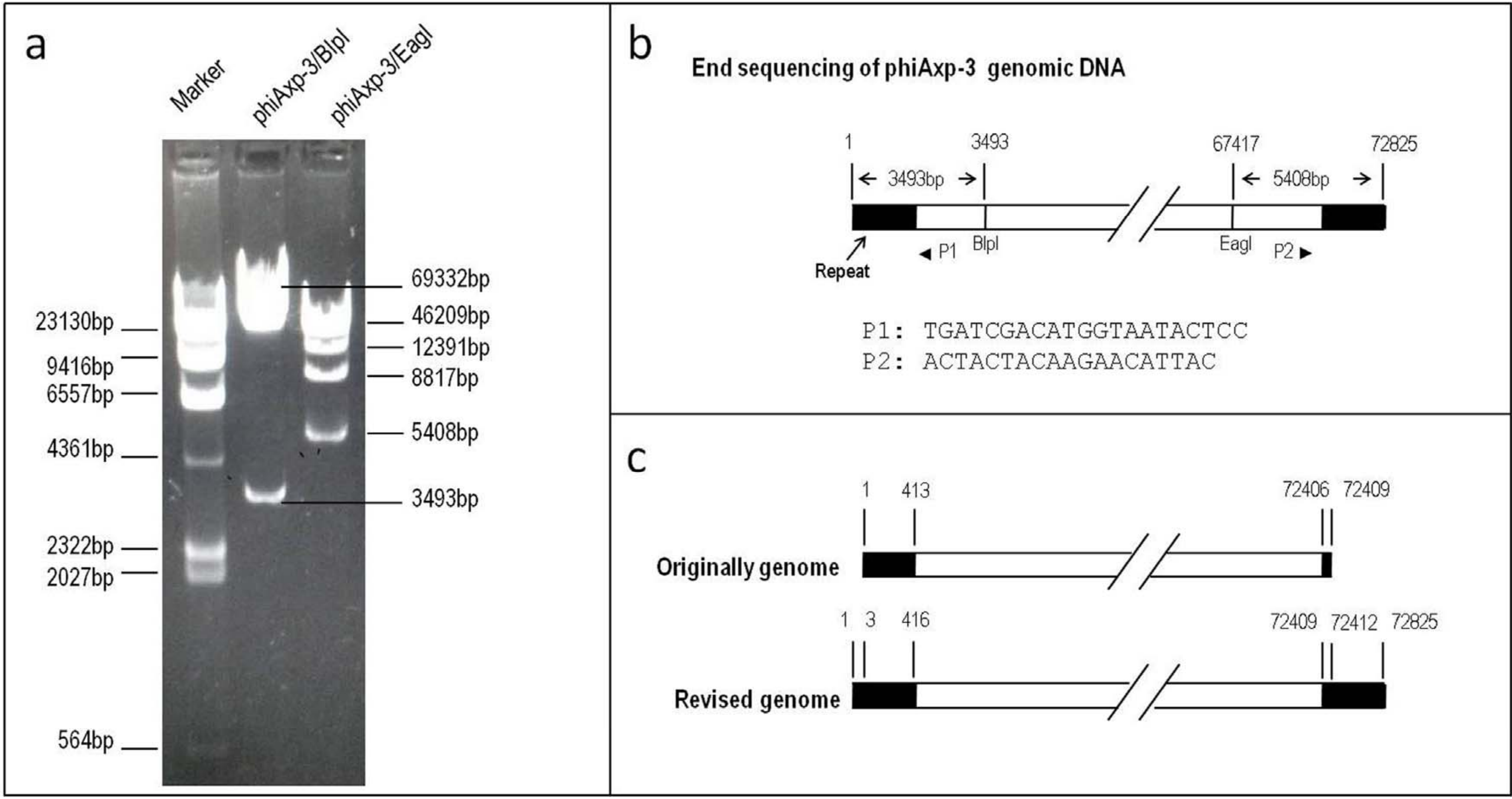

a
Marker
phiAxp-3/BlpI
phiAxp-3/EagI
23130bp
9416bp
6557bp
4361bp
2322bp
2027bp
564bp
69332bp
46209bp
12391bp
8817bp
5408bp
3493bp
b
End sequencing of phiAxp-3 genomic DNA
1
3493
67417
72825
3493bp
5408bp
Repeat
P1
BlpI
EagI
P2
P1: TGATCGACATGGTAATACTCC
P2: ACTACTACAAGAACATTAC
c
1
413
72406
72409
Originally genome
1
3
416
72409
72412
72825
Revised genome

```
JWAlpha         -----------------------------ACACACCCCCCGGTGTCTTGCTCCTGTGCAC
JWDelta         -----------------------------------CCCCCGGTGTCTTGCTCCTGTGCAC
phiAxp-3        TATGTGTACACGGTACACCCTACACTTTATACACCCCCCCGGGGTCTCTC-CTAGCCCAC
                                                   ******* ****  * *  *  ***

JWAlpha         CTCTACCCA--CCCCCATGTTGTTGACGCTCCGCGTCTTGTGGGTCTGTTGCCTATCATC
JWDelta         CTCTACCCA--CCCCCATGTTGTTGACGCTCCGCGTCTTGTGGGTCTGTTGCCTATCATC
phiAxp-3        ACCTACCCTACCCCCCATGTTTCTGACGCTCCGCGTCTTCTGGGTATGTTGCC---CATC
                  ******   **********  **************** ***** *******   ****

JWAlpha         TATCAATCTTCTTAGGAGTATTAAC-ATGTCCACTTCCACCGCTCGCATGACCATCGGCT
JWDelta         TATCAATCTTCTTAGGAGCATTAAC-ATGTCCACTTCCACCGCTCGCATGACCATCGGCT
phiAxp-3        TATC--TTTCTTTAGGAGTATCAACTATGTCCACGTCCACCGCTCGCCTGACCATCGGCT
                ****  * *  ******* ** *** ******** ************ ************

JWAlpha         CTGCTCTCGGTACGATCAATACCGCTGCGCTTACCATCTCGTCCACTCTGGATGCAGTTG
JWDelta         CTGCTCTCGGTACGATCAATACCGCTGCGCTTACCATCTCTTCCACTCTGGATGCAGTTG
phiAxp-3        CTCTGCTCGGTACCGTGAATACTGCCGCCATCACCGTGTCGTCCACTCTGGATGCTGCTG
                **   ********  * ***** ** **  * *** * ** ************** * **

JWAlpha         GCTCTGGTGTTGGTATGCTCAACGCCATGGTTGACAAGGCTGCTCGTGAACAACGTATGC
JWDelta         GCTCTGGTGTTGGTATGCTCAACGCCATGGTTGACAAGGCTGCTCGTGAACAACGTATGC
phiAxp-3        GTGCTGCTGTCGGTATGCTCAACTCTGCTGTCTCCAAGGCAGCTAAAGAGCAAGCTATCC
                *  *** *** ************ *    **   ****** ***   ** ***  *** *

JWAlpha         GTCACAAAGCTGACGTCTCCGAGTTCAAGCACCGTCTCATCAACGAAGTAGCTATGGCTC
JWDelta         GTCACAAAGCTGACGTCTCCGAGTTCAAGCACCGTCTCATCAACGAAGTAGCTATGGCTC
phiAxp-3        GTCACAAGGTTGACGTCAACGAGTTCAAGAGTCGCCTGATCATGGAAGTCTCCATGGCTC
                ******* * *******  **********   ** ** ****  *****  * *******

JWAlpha         GTGCACAACGTGAACGTCAAGTCATAGAGTTCTGCA------------------------
JWDelta         GTGCACAACGTGAACGTCAAGTCATAGAGTTCTGCAAGGACAAGGACAATGAAACCTTGT
phiAxp-3        GTGCTCAACGTGAGCGTCAAGTAGTAGAGTTCTGCAAGGACTCTGTCAACGAGAAGCTCT
                **** ******** ********  ************

JWAlpha         --------------------------------------
JWDelta         ACTCCAAGGCTCACGACGAACTCACCGAGTTGCTCAAG
phiAxp-3        TC------------------------------------
```
